# Microarray karyotyping of commercial wine yeast strains reveals shared, as well as unique, genomic signatures

**DOI:** 10.1186/1471-2164-6-53

**Published:** 2005-04-16

**Authors:** Barbara Dunn, R Paul Levine, Gavin Sherlock

**Affiliations:** 1 Dept. of Genetics, Stanford University Medical Ctr., Stanford, CA 94305-5120, USA; 2 Dept. of Biological Sciences, Stanford University, Stanford, CA 94305-5020, USA

## Abstract

**Background:**

Genetic differences between yeast strains used in wine-making may account for some of the variation seen in their fermentation properties and may also produce differing sensory characteristics in the final wine product itself. To investigate this, we have determined genomic differences among several *Saccharomyces cerevisiae *wine strains by using a "microarray karyotyping" (also known as "array-CGH" or "aCGH") technique.

**Results:**

We have studied four commonly used commercial wine yeast strains, assaying three independent isolates from each strain. All four wine strains showed common differences with respect to the laboratory *S. cerevisiae *strain S288C, some of which may be specific to commercial wine yeasts. We observed very little intra-strain variation; i.e., the genomic karyotypes of different commercial isolates of the same strain looked very similar, although an exception to this was seen among the Montrachet isolates. A moderate amount of inter-strain genomic variation between the four wine strains was observed, mostly in the form of depletions or amplifications of single genes; these differences allowed unique identification of each strain. Many of the inter-strain differences appear to be in transporter genes, especially hexose transporters (HXT genes), metal ion sensors/transporters (CUP1, ZRT1, ENA genes), members of the major facilitator superfamily, and in genes involved in drug response (PDR3, SNQ1, QDR1, RDS1, AYT1, YAR068W). We therefore used halo assays to investigate the response of these strains to three different fungicidal drugs (cycloheximide, clotrimazole, sulfomethuron methyl). Strains with fewer copies of the *CUP1 *loci showed hypersensitivity to sulfomethuron methyl.

**Conclusion:**

Microarray karyotyping is a useful tool for analyzing the genome structures of wine yeasts. Despite only small to moderate variations in gene copy numbers between different wine yeast strains and within different isolates of a given strain, there was enough variation to allow unique identification of strains; additionally, some of the variation correlated with drug sensitivity. The relatively small number of differences seen by microarray karyotyping between the strains suggests that the differences in fermentative and organoleptic properties ascribed to these different strains may arise from a small number of genetic changes, making it possible to test whether the observed differences do indeed confer different sensory properties in the finished wine.

## Background

Winemakers have long noted that different strains of wine yeasts, even when used to ferment the same juice under identical conditions, can yield very different wines in terms of sensory characteristics [1,2], presumably as a result of variations in the strains' fermentative properties [3-7]. Previous studies have demonstrated genetic diversity among both commercial [8] and wild [9]*Saccharomyces cerevisiae *wine yeast strains, and it has been hypothesized that this genetic diversity may, at least in part, be a root cause of their differing fermentative and sensory qualities [10-16]. In addition to a large amount of diversity in mitochondrial DNA [17], some wine yeast strains have been shown to be genetically unstable [18,19], with varying ploidy levels [20,21], multiple chromosomal aneuploidies [21-23], and chromosome length polymorphisms [21,22,24-26]. In addition, the genomes of a few wine yeast strains appear to have arisen from a hybridization event between two species, *S. cerevisiae *and *S. bayanus*[27], similar to that of the lager yeast *S. pastorianus *[28].

Many studies have shown that extensive genome rearrangements occur in organisms during adaptation to changing environments [29-31]. It is also likely that any source of cellular stress, such as an environmental insult, may trigger a "genome shock" response [32,33], leading to genome rearrangements. Although some genome changes observed in yeast populations are on a small scale (one to a few nucleotides), many changes appear to be on the scale of entire genes or even whole chromosomes, resulting in alterations of their copy numbers [31,34]. These features may be a result of uneven mitotic crossing over [35,36], gene conversion or break-induced replication [18,37], or Ty transposon-mediated chromosomal translocations[25].

Wine-making has been an ongoing human activity for several millennia [38-40]. Although the wine strains we have examined in this paper are commercial strains that have been propagated industrially for only the last ~40 years, each of their progenitors were distinct wild strains that had presumably been selected for many hundreds, perhaps thousands, of years to confer specific organoleptic traits. Likewise the laboratory S288C strain has been propagated as a pure culture for ~70 years; while its exact background is unknown and may include both wild and baking yeasts, it is almost certain that S288C did not derive from any commonly-used wine yeast strains [41]. At a generation time of 1.5 hours, however, 70 years can represent thousands (possibly hundreds of thousands) of generations of divergence between commercial wine yeasts and their laboratory "cousins". The genomic differences that exist among these different wine yeast strains, and between wine and laboratory yeasts, may have arisen in response to the unique selective pressures that each has encountered during its propagation. Detailed characterization of these differences may thus shed light on which biochemical pathways and cellular processes play important roles in determining the specific fermentative qualities (and the resultant wine sensory characteristics) of a particular wine yeast strain.

The recent development of DNA microarrays has enabled an unprecedented level of genome-scale research on both gene expression patterns [42,43], and also on genomic DNA copy number changes and genome rearrangements through the "array-CGH" ("aCGH") technique [44,45], which we will refer to as "microarray karyotyping" or "array karyotyping". This technique can be used to determine copy number changes for every gene of a given species (whose genome has been sequenced) in relation to any other strain of that species, giving information on whole or partial chromosome aneuploidies, non-reciprocal translocations and isolated gene deletions or amplifications. Using human cDNA microarrays, this technique has helped elucidate genomic copy number changes in cancer cells during tumor progression [46-48]; using *S. cerevisiae*-based microarrays, it has also been employed to discover non-reciprocal chromosomal translocations that occurred in yeasts evolved to tolerate low glucose concentrations [31]. A number of previous papers have demonstrated that aCGH data accurately reflects genome changes. For example, Pollack et al.[45], using human cDNA microarrays, demonstrated good correlation of copy number of known human X-chromosome aneuploid lines (from 0 to 5 X chromosomes per diploid genome) with aCGH data. Likewise for yeast microarrays, standard PCR [49,50] or quantitative real-time PCR [51] has been used to validate either deletions or amplifications predicted by aCGH data. Also, Dunham et al. [31] and Winzeler et al. [52] have used DNA sequencing to validate rearrangements (with associated copy number changes) or single-nucleotide polymorphisms, respectively, to corroborate their aCGH results.

Until recently, studies of the genetic diversity and instability of wine yeasts (reviewed by [8,50]) were performed using either classical genetic means, pulsed-field gel analysis, or by molecular techniques such as PCR, Southern blotting and restriction-fragment length polymorphisms. These studies did not, however, utilize whole-genome platforms such as microarrays, and thus the results were confined to only certain regions of the genome. Global gene expression patterns in wine yeast strains, deduced by microarray analysis, have been recently reported [53-55], but these studies did not include microarray karyotyping of the wine yeasts.

Microarray karyotyping has also been used to explore the genomic diversity of different *S. cerevisiae *strains [50,52,56-58] as well as the genomic architectures (relative to *S. cerevisiae*) of the hybrid organism *S. pastorianus *[51] and the *sensu stricto *group of closely-related *Saccharomyces *species [59]. However, none of these studies have utilized microarray karyotyping to assay the genomes of commercially-used wine yeasts for both copy number increases and decreases relative to the sequenced S288c laboratory strain. As part of larger gene expression studies, Lashkari et al. [57] and Primig et al. [58] generated array karyotype data, relative to S288C, for the non-S288C-derived laboratory *S. cerevisiae *strains Y55 and SK1 respectively; however these authors did not analyze any wine strains by array karyotyping. Filter-based whole-ORF genome arrays have also been used to investigate global gene expression patterns in a wine yeast [60]. These authors also re-hybridized a filter array using labeled genomic DNA to obtain gene copy numbers changes in the wine strain relative to the laboratory strain, but the data as presented did not show many interpretable changes in the wine strain other than a decrease in Ty1 copy number relative to the laboratory strain; a small amount of the same data generated by [60], restricted to chromosome VIII, was also presented in a separate paper [50].

Winzeler et al. [52] performed a type of array karyotyping on the Y55 strain, as well as on a wine strain, by using high-density short (25-mer) oligo arrays; however, their technique only allowed the detection of complete deletions at the whole gene or chromosome level, i.e., they could not detect amplification or hemizygous depletion of genes or chromosomes (e.g., the increase or decrease of just one copy of a gene or chromosome in a diploid cell, giving either three or one copies, respectively). It is important to note that whole-ORF arrays allow hybridization of even a fairly diverged gene (20 – 25% nucleotide divergence) to the target ORF on the microarray due to the long length of the target. Such divergence is much more likely to occur in non-S288C strains such as wine strains. 25-mer oligo arrays, however, are exquisitely sensitive to single-nucleotide changes; this is useful in some regards, but less so when widely-diverged strains are being analyzed. Infante et al. [56] performed a very detailed microarray karyotyping study of the genomic differences between two "flor" yeasts used in the production of sherry wines; however, they only compared the two "flor" yeasts to each other, and not to the sequenced S288C laboratory strain. Finally, both Edwards-Ingram et al. [59] and Bond et al. [51] used *S. cerevisiae *spotted ORF microarrays to look at the whole genome organization of other species that are closely related to *S. cerevisiae*, but did not assess the genomes of wine yeasts.

We describe here the use of microarray karyotyping to explore global changes in the genomic DNA of four commonly-used commercial wine *S. cerevisiae *strains relative to each other and to the sequenced *S. cerevisiae *laboratory strain (S288C), including the analysis of three independent isolates of each of the four wine strains. We report on the characterization of genomic similarities and differences between the wine strains and the S288C laboratory strain, as well as between the different wine strains themselves and between different independent isolates of the same strain.

## Results and discussion

### Microarray-based karyotyping

In the microarray karyotyping protocol employed here, genomic DNA from an experimental strain (i.e., a commercial wine yeast strain) was labeled with the fluorescent dye Cy5 (red), while genomic DNA from a wild-type (WT) reference strain (the sequenced haploid laboratory *S. cerevisiae *strain S288C) was labeled with the fluorescent Cy3 dye (green). The two labeled samples were mixed, then competitively hybridized to a spotted DNA microarray in which each spot is composed of PCR-amplified DNA corresponding to one gene of the reference S288C yeast *S. cerevisiae *[31,61]. Under standard conditions [31,61], hybridization to the microarray spots will generally occur between DNA molecules with 75% or more sequence identity. All experiments were performed in duplicate, i.e., the labeled DNA was hybridized to two separate microarrays. Note that the raw data for all microarray hybridizations reported in this paper can be downloaded from the website  as well as from the Stanford Microarray Database [62,63].

Assuming that the experimental strain's genome has not experienced significant sequence divergence (i.e., greater than 25%) from that of the laboratory reference strain – a supposition which appears to be true for most *S. cerevisiae *wine yeast strains, which have an average of greater than 99% identity to the S288C strain [64] – any DNA copy number change in a single gene within the experimental strain will be detected by a deviation of the R/G ratio for that gene from the expected 1:1 ratio. If there are proportionally more copies of the gene in the experimental strain relative to the reference strain, the R/G ratio will thus be higher than 1:1 (ideally, it will be 2:1 for a duplication event, 3:1 for a triplication, etc.). Likewise, if there has been a deletion of the gene in the experimental strain relative to the reference strain, or if there are more copies of the gene in the reference strain than in the experimental strain (i.e., the experimental strain has experienced a "depletion" of the gene in terms of copy number), the R/G ratio will be less than 1:1 (ideally, it will be 1:2 for a single duplication in the reference strain relative to the wine strain, or for a deletion in the experimental strain of a single-copy gene in the reference strain, and so on). Thus, with only one hybridization, the DNA copy number of all 6,000 genes of a given strain can be determined relative to the reference strain.

Because microarray karyotyping detects proportional changes in DNA copy number relative to a reference strain, genome rearrangements that result in net copy number alterations can be easily detected with this method, including gene duplications or deletions, as well as larger-scale rearrangements such as non-reciprocal translocations, loss or amplification of large chromosomal regions, and ploidy changes for a single entire chromosome (aneuploidy). These large-scale rearrangements are seen as large regions of the chromosomes for which a similar bias in the ratios exists for all the genes in the region. Thus, despite the fact that complete genomic sequencing of every wine yeast strain is impractical, a great deal of information about the genomic structure of wine strains relative to laboratory strains and to each other can be achieved by microarray karyotyping.

### Strains used for analysis

We chose four different commercial wine yeast strains for this study, using independent isolates from each of three different commercial and/or academic sources as shown in Table 1. The four yeast strains used were Montrachet, Pasteur Red, Pasteur Champagne, and Prise de Mousse (see Table 1 and Methods for complete list). These are among the most commonly used commercially-produced yeast strains in winemaking. Each strain has distinct fermentative qualities, and each is thought to bring unique flavor and other sensory components to the finished wine product [65,66]. Independent isolates of each strain were obtained from two major wine yeast producers, Lalvin and Red Star, as well as from the wine yeast strain collection at Univ. of Calif., Davis (Table 1).

**Table 1:** Wine Yeast Strains used in Study

***Wine Yeast Type *^*a*^**	***Lab Name *^*b*^**	***Product Name *^*c*^**	***Source *^*d*^**	***Wine Use *^*e*^**
				
1. Montrachet	GSY1	Montrachet	Lalvin	White, Red
	GSY2	Montrachet	Red Star	
	GSY3	Montrachet (UCD522)	U.C. Davis	
2. French Red	GSY4	Pasteur Red	Lalvin	Red
	GSY5	French Red (UCD725)	U.C. Davis	
	GSY6	French Red (UCD904)	U.C. Davis	
3. Past. Champagne	GSY7	Champagne	Lalvin	Sparkling
	GSY8	Past. Champ.	Red Star	
	GSY9	Past. Champ. (UCD595)	U.C. Davis	
4. Prise de Mousse	GSY10	EC1118	Lalvin	White, Red
	GSY11	Premier Cuvee	Red Star	
	GSY12	Prise de Mousse (UCD819)	U.C. Davis	

^*a*^*General category of wine yeast. ^b^Name used in laboratory collection. ^c^Name used by source (manufacturer); numbers in parentheses indicate the U.C. Davis strain collection name. ^d^Commercial source of strain; see Methods for contact information. ^e^General type(s) of wines for which strains are used*.

### The genomes of all four wine yeast strains are highly similar to that of *S. cerevisiae *S288C

We performed microarray karyotyping, as described in Methods, on each of the wine yeast strains. Because some of the strains are listed by suppliers as either *S. bayanus *or *S. cerevisiae/S. bayanus*, we also performed microarray karyotyping experiments using genomic DNA from a known type strain of *S. bayanus *to determine what possible signal may be generated when hybridizing *S. bayanus *genomic DNA to *S. cerevisiae *microarrays. We performed a similar experiment using *S. cerevisiae *DNA as a control (a "self-self" hybridization).

**Figure 1:**
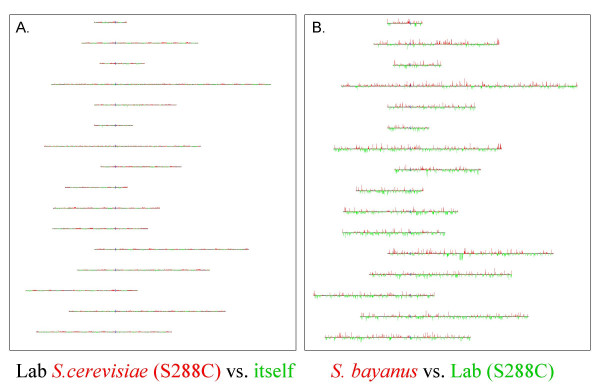
**Karyoscope views of S288C-S288C and *S. bayanus*-S288C microarray hybridizations. **Microarray hybridizations were performed as described in the text. In both panels the *S. cerevisiae *laboratory strain S288C was used as a reference sample, labeled with Cy3 dye (green). In panel A, the Cy5-labeled (red) DNA was also S288C, giving a self-self hybridization. In panel B, the Cy-5 labeled DNA was *S. bayanus*. The labeled DNAs were competitively hybridized to spotted microarrays bearing full-length ORFs from the S288C *S. cerevisiae *strain; the data thus obtained is displayed here in graphical form as a karyoscope. Red bars indicate red:green ratios above 1.0 and are graphed on a log scale; green bars indicate red:green ratios below 1.0, also graphed on a log scale. The chromosomes are shown in each panel in numerical order with chromosome 1 at the top and chromosome 16 at the bottom, and are aligned by their centromeres, with their left arms extending to the left. Both karyoscopes are drawn to the same scale, i.e., the bar heights in panels A and B proportionally represent the same amplitude of change.

Figure 1 shows a graphical representation of the microarray karyotyping data for the *S. cerevisiae *and *S. bayanus *strains presented as "karyoscope" diagrams, made using the Java TreeView program [67]. To make a karyoscope diagram the hybridization ratio for each gene is mapped onto its corresponding position on each chromosome of the reference strain of *S. cerevisiae*. The height of each red or green vertical bar is proportional to the log_2 _of the red:green (R/G) ratio for a gene; if the ratio is greater than 1 (i.e., a positive log_2 _value), the bar will be red and drawn above the chromosome; a red bar thus represents an over-representation (amplification) of that gene in the wine strain relative to S288C. For R/G ratios less than 1 (i.e., a negative log_2 _value), the bar will be green and drawn below the chromosome; a green bar thus represents an under-representation (deletion or depletion, i.e., lower copy number) of that gene in the wine strain relative to S288C.

In the "self-self" hybridization control, the genomic DNA from the reference *S. cerevisiae *laboratory strain was labeled independently with Cy3 and Cy5 and then competitively hybridized against itself. We observed robust hybridization across the entire array, and after a global mean normalization (see Methods) there were equal red and green hybridization signals (R/G ratios of 1), with no obvious deviations for any particular gene, as expected (Fig. 1A). We found, however, that the *S. bayanus *DNA did not hybridize to the arrays in the same manner as did the *S. cerevisiae *genomic DNA. The *S. bayanus *hybridization signals (Fig 1B) were significantly weaker and much more variable across the array, as compared to the *S. cerevisiae *self-self hybridization (Fig 1A).

For all 12 of the wine yeast isolates, we observed hybridization patterns much more similar to that of the "self-self" experiment, i.e., with robust signals across most of the array, and the majority of ratios near a value of 1.0. This indicates that each of the wine strains possesses an essentially complete complement of the *S. cerevisiae *genome, with no observed aneuploidy. Note that we cannot rule out that there may be additional genomic DNA in these wine strains that comes from other *Saccharomyces *species (i.e., if the strain is a hybrid organism) whose hybridization signal would be overshadowed by the signal generated by the *S. cerevisiae *genomic complement. However, we do not think this is likely, as control spots on the microarrays containing *S. bayanus *genomic DNA did not show a hybridization signal, which we consistently observe when using known *S. cerevisiae – S. bayanus *hybrid yeasts (B. Dunn, unpublished). Thus, we do not expect any potential non *cerevisiae *DNA in any of these wine strains to have any great effect on our observed ratios.

### Genomic differences between wine yeast strains and the laboratory strain

**Figure 2:**
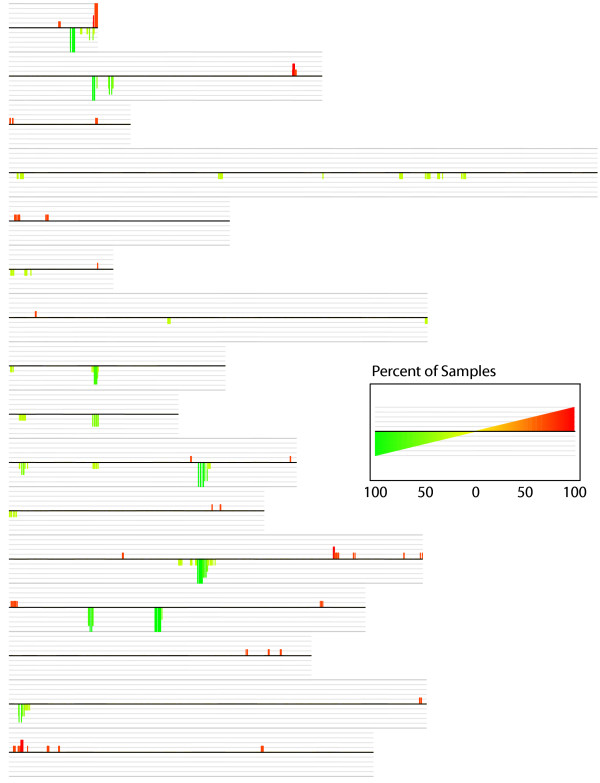
**Changes in Gene Copy Number Shared by all Wine Strains: the "Commercial Wine Yeast Signature"**. A consensus plot pooling the results of all the wine strains relative to the lab strain S288C was generated by the program CGH-Miner [68]. This plot is similar to a karyoscope in that it displays values along each chromosome, and the chromosomes are shown in numerical order from top to bottom. However, in this plot, a determination of which regions were statistically significantly altered in copy number for each wine strain (i.e., averaged array values for each of the three isolates within a strain) relative to the laboratory S288C strain were determined. For each of these regions the number of wine strains showing a significant change were then added together to give a metric of how well-shared among the wine strains that particular change was. This is shown as tall red bars for significantly amplified regions among all wine yeast strains relative to the laboratory strain, and as tall green bars for significantly depleted regions in all the wine strains relative to the laboratory strain. Each gray "shadow line" above or below the chromosome represents 20% of the number of wine strains showing a significant change in that region (see legend). The group of changes comprising the tallest (>75%) red and green bars represents the "commercial wine strain signature"; these are listed in Table 2.

We observed a distinct set of genomic variations that occur in every wine strain relative to the laboratory strain; these are shown in Figure 2, and an abbreviated list of these variations is given in Table 2. Figure 2 represents a consensus karyoscope-type plot generated by the program CGH-Miner [68], where the data from the 4 wine strains (taken as averages of the data for the 3 isolates of each strain) have been consolidated into statistically-significant amplifications or depletions relative to multiple self-self hybridizations of the laboratory S288C strain.

**Table 2:** "Commercial Wine Strain Signature" Gene List^*a*^

**Sig. amplified in at least 3 strains**		**Montrachet**	**French Red**	**Champagne**	**Prise de Mousse**
YAR066W		**0.84**	**1.43**	**0.62**	*0.19*
YAR068W		**1.05**	**1.67**	**0.91**	**0.52**
YAR069C		**1.11**	**1.57**	**1.09**	**0.59**
YAR070C		**1.14**	**1.62**	**1.16**	**0.63**
YAR071W	PHO11	**1.01**	**1.37**	**1.26**	**0.57**
YAR073W	IMD1	**0.99**	**1.41**	**1.34**	**0.56**
YAR075W		**0.98**	**1.47**	**1.46**	**0.56**
YPL276W		*0.59*	**1.36**	**1.64**	**0.75**
YPL275W	FDH2	*0.63*	**1.39**	**1.68**	**0.78**
YPL274W	SAM3	*0.58*	**1.06**	**1.31**	**0.66**
YPL273W	SAM4	*0.52*	**0.81**	**1.04**	**0.57**
YPL272C		*0.49*	**0.59**	**0.79**	**0.47**
					
**Sig. depleted in at least 3 strains**		**Montrachet**	**French Red**	**Champagne**	**Prise de Mousse**
YAR007C	RFA1	**-0.44**	**-0.73**	*-0.80*	**-0.31**
YAR008W	SEN34	**-1.05**	**-1.53**	**-1.31**	**-0.69**
YAR014C	BUD14	**-1.03**	**-1.25**	**-0.99**	**-0.65**
YAR015W	ADE1	**-0.54**	**-0.63**	**-0.64**	**-0.38**
YBL007C	SLA1	**-0.51**	**-0.61**	**-0.81**	**-0.39**
YBL006C	LDB7	**-0.86**	**-0.99**	**-1.02**	**-0.66**
YBL005W	PDR3	**-0.88**	**-1.07**	**-1.10**	**-0.67**
YBR013C		**-0.97**	**-1.58**	**-1.04**	*-0.45*
YHR052W	CIC1	*-0.39*	**-0.89**	**-1.40**	**-0.78**
YHR053C	CUP1-1	*-0.46*	**-1.32**	**-1.91**	**-1.07**
YHR054C		*-0.56*	**-1.65**	**-2.16**	**-1.29**
YHR055C	CUP1-2	*-0.56*	**-1.43**	**-1.98**	**-1.26**
YHR056C	RSC30	*-0.49*	**-1.10**	**-1.52**	**-0.96**
YJR024C		**-0.59**	**-0.62**	**-0.49**	**-0.30**
YJR025C	BNA1	**-0.91**	**-1.13**	**-0.84**	**-0.53**
YJR026W		**-1.47**	**-1.92**	**-1.41**	**-0.90**
YJR030C		**-0.95**	**-1.65**	**-1.32**	**-0.67**
YJR031C	GEA1	**-0.41**	**-0.90**	**-0.78**	**-0.32**
YJR032W	CPR7	**-0.16**	**-0.31**	**-0.39**	*-0.15*
YJR033C	RAV1	**-0.14**	**-0.32**	**-0.39**	*-0.11*
YML047C	PRM6	**-0.46**	**-0.89**	**-0.91**	*-0.25*
YML046W	PRP39	**-0.51**	**-1.16**	**-1.19**	*-0.31*
YML043C	RRN11	**-0.49**	**-0.99**	**-1.05**	*-0.33*
YML042W	CAT2	**-0.83**	**-1.33**	**-1.16**	**-0.51**
YML041C	VPS71	**-0.93**	**-1.35**	**-1.18**	**-0.58**
YML038C	YMD8	**-0.84**	**-1.39**	**-1.16**	*-0.46*
YML037C		**-0.47**	**-0.97**	**-0.93**	*-0.23*
YMR043W	MCM1	**-0.30**	**-0.28**	**-0.30**	**-0.23**
YMR044W	IOC4	**-0.81**	**-0.98**	**-0.82**	**-0.59**
YMR046W-A		**-1.14**	**-1.58**	**-1.29**	**-0.78**
YMR047C	NUP116	**-0.70**	**-0.96**	**-0.88**	**-0.56**
YMR048W	CSM3	**-0.69**	**-0.89**	**-0.75**	**-0.46**
YMR049C	ERB1	**-1.07**	**-1.45**	**-1.03**	**-0.66**
YMR052W	FAR3	**-1.17**	**-1.87**	**-1.28**	**-0.63**
YMR052C-A		**-0.69**	**-1.26**	**-0.89**	**-0.36**
YOL165C	AAD15	**-1.09**	**-1.03**	*-0.74*	**-0.80**
YOL164W		**-1.33**	**-1.24**	*-0.96*	**-0.80**

^*a *^*The data shown in this table are the actual mean log_2_(R/G) ratios for each gene, calculated by averaging the values from the six arrays (duplicate arrays of each of the three isolates of each strain) hybridized per wine strain. This list represents the wine-specific subset of the total set of genes with significant copy number changes, as described in the text. Values shown in bold text were determined by the CGH-Miner program to be significantly depleted or amplified as indicated. Values not significantly depleted or amplified are shown in non-bold italicized text*.

The most distinctive genomic variations seen in Fig. 2 are the many prominent green bars which are seen on most of the chromosomes. These shared green bars represent genes or regions in the wine strains, taken together as a whole, that are depleted in copy number relative to the laboratory strain. In most cases these shared depleted regions consist of genes that are moderately or highly repeated in the laboratory strain, with the majority representing Ty1 elements (a yeast retrotransposon family). Also depleted are the tandemly-repeated *ASP3 *(asparaginase) genes on chromosome 12, and the tandemly-repeated *ENA *(sodium transport) genes on chromosome 4; note: general descriptions and functions for genes described in this paper were obtained from the *Saccharomyces *Genome Database (SGD) [69,70]. The *CUP1 *(copper binding) tandemly-repeated genes are also significantly depleted in 3 of the 4 strains (Fig. 2, Table 2) and additionally show some intra-strain variation in copy number (Fig. 5B); depletion of the *CUP1 *genes has been observed previously in a wine strain [50]. Because these sequences are all moderately repetitive and highly conserved, we can only interpret these results to indicate that in all of the wine strains we investigated, there are either fewer or no copies of these repeated genes relative to the laboratory strain.

Because the CGH-Miner program utilizes a moving-average method for calculating significance, copy number changes of single isolated genes are not detected. However, inspection of clustered data and karyoscopes allowed us to identify single genes whose copy numbers are highly altered relative to the reference strain. Among these, we found that the single-copy drug response genes *SNQ2 *and *PDR15 *are depleted in all of the wine strains we examined (Fig 5B). In addition, *PDR3*, a single-copy zinc-finger transcription factor involved in pleiotropic resistance to drugs [71], appears to be entirely deleted in all four wine strains due to the very low R/G ratios it exhibits (Table 2, Fig. 5B). This gene resides adjacent to one of the Ty1 elements that is depleted in the wine strains. Additional altered-copy number genes identified by inspection of karyoscopes are listed in Fig. 5B.

In addition to the shared depleted regions, there are shared regions that appear to be slightly, but consistently, amplified in all four wine yeast strains; the most prominent is located at the right end of chromosomes 1; other regions are on the right arms of chromosomes 2 and 12, and the left end of chromosome 16 (Fig. 2, Table 2). These shared amplified regions contain some genes that are members of homologous gene families in the reference strain, such as the *IMD *genes (involved in GMP production) and *PHO *genes (acid phosphatases), but they also include unique genes such as *SAM3 *and *SAM4 *(S-adenosyl methionine transport and S-AM metabolism, respectively), *PET8 *(mitochondrial S-AM transport), *RDS1 *(response to xenobiotic drugs, including fungicides, herbicides, and pesticides), *TPO1 *(multidrug and polyamine transporter and drug detoxification), *AAD3 *(aryl-alcohol dehydrogenase), *ADH7 *(alcohol dehydrogenase), and *FDH1 *and *FDH2 *(formate dehydrogenase) (Table 2, Fig. 5B). Also occurring in an amplified region is the un-named and uncharacterized gene *YAR068W *(Table 2, Fig. 5B). This gene codes for a membrane protein which when overexpressed results in resistance to the drug ketoconazole [72]; additionally, it has been found to have one of the most elevated K_a_/K_s _ratios among the genomes of the *Saccharomyces sensu stricto *group [73], signifying that it may be a gene that is actively undergoing positive selection [74]. A homologue to this gene, *YHR214W-A*, is also amplified in most of the wine yeast strains.

These results indicate that all of the wine strains that we examined have experienced a characteristic increase or decrease in copy number, relative to that of the laboratory strain, of the genes listed in Table 2. Although some of these changes, particularly the low copy number (or absence) of the *ENA *and *ASP3 *genes and of Ty1 elements, are consistently observed in other industrial and non-S288C laboratory yeast strains ([51,52,57-60], B. Dunn, unpublished), most of the other changes, particularly the amplifications, are novel and thus far appear to be unique to commercial wine yeasts.

### Genes whose copy number changes are shared by all wine strains

Interestingly, there are many transporter and permease genes, including some involved in drug response, that show altered copy numbers in the all the wine strains relative to the laboratory strain. As described above, *PDR3, PDR15, RDS1*, *SNQ2, TPO1*, and *YAR068W *show alterations in their copy numbers that are shared by all the wine strains. In addition, *HXT9 *and *HXT11 *are depleted in three of the four strains (Fig. 5B), and *AYT1 *is depleted in 2 strains and amplified in the other two. All of these genes are involved in some aspect of drug response [71,72,75-78].

It thus appears that a major group of shared genomic differences found among all the wine strains is composed of genes, especially transporters and permeases, involved in drug resistance pathways. Since vineyards are often treated with herbicides, pesticides, and fungicides, wine yeasts may be exposed to these agents on a routine basis and their genomes may have evolved to better tolerate such exposure. Alternatively – and perhaps more likely given the fact that these commercial strains may have been isolated prior to the widespread use of herbicides, pesticides and fungicides – the variations in transporter gene copy number seen here may have arisen to fine-tune the fermentation properties of the wine yeasts by altering the types and amounts of fermentable substrates that are taken up. These variations may also reflect an increased tolerance to, or excretion of, plant phenolic compounds. Thus, any changes in drug response pathways in these strains may be a secondary effect and not due to direct selection for drug resistance or sensitivity. This view is supported by reports that alterations in *PDR1 *and *PDR3 *gene expression, as well as mutations in the ABC transporter genes that they regulate, result in better fermentation performance by sake yeasts [79,80].

### Inter-strain variation

**Figure 3:**
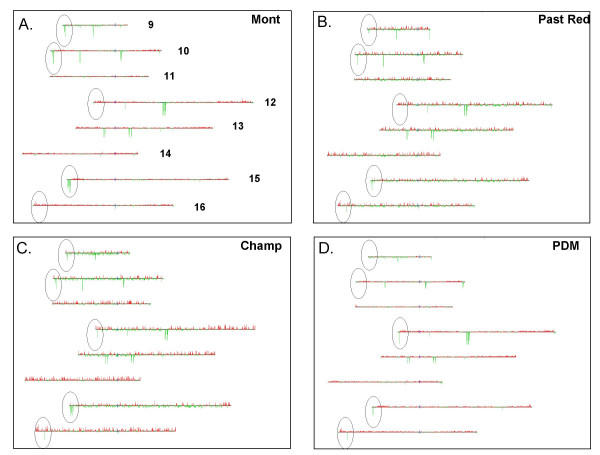
**Wine strain karyoscopes. **Microarray ratios from each of the three isolates of a given wine strain were averaged to yield an "average" microarray karyotype for each wine strain. Chromosomes 9 through 16 for the 4 strains are shown in panels A – D as follows: A. "Mont" = Montrachet (average of strains GSY1-3). B. "PDM" = Prise de Mousse (average of strains GSY10-12). C. "Champ" = Pasteur Champagne (average of strains GSY7-9). D. "Red" = French Red (average of strains GSY4-6). Circled regions highlight areas that vary in characteristic ways between the different wine strains. All karyoscopes are drawn to the same scale.

We also observed inter-strain genomic variations that are specific to each wine strain. Examples of such inter-strain genomic variation are highlighted as regions circled in black on the Karyoscopes in Figure 3A–D. For an idea of scale in these karyoscopes, the tallest bars on the figure represent ratios of approximately 2:1 (2-fold enhanced) for red, and 1:16 (16-fold depleted) for green. Each panel in Fig. 3 represents the average of the hybridization log ratios for all three independent isolates of the given strain; since each isolate's DNA was hybridized in duplicate, each panel thus represents the average of six arrays, making the data very robust. Some examples of inter-strain variation are also shown in more detail in Figure 5A, in which the hybridization data are represented as colored boxes, with the most intense green representing severe depletion (R/G ratios much less than 1), black representing no change in copy number (R/G ratio of approximately 1), and the most intense red representing significant amplification (R/G ratios greater than 1).

One distinct inter-strain variation is seen in the copy numbers of *HXT *(hexose transporter) genes. As shown by the black-circled regions on chromosomes 9 and 10 in Fig. 3A–D, and in the top panel of Fig. 5A, the Prise de Mousse isolates show no depletion of the *HXT9*, *HXT11*, and *HXT12 *genes, whereas the Pasteur Red isolates show a slight depletion of these genes. On the other hand, the Montrachet and Champagne isolates show extreme depletion of these genes. Since these three *HXT *genes are all virtually identical to each other and can cross-hybridize, this result is most likely explained by a loss of one or more of the three genes in the depleted strains. It is impossible, however, to know which particular gene(s) are missing without further investigation.

Another class of inter-strain differences is of particular interest because the differences are seen in genes that are single-copy in the laboratory strain. Depletions of these genes in a given wine strain as measured by array karyotyping may thus reflect a true deletion of that gene relative to the other wine strains, rather than just a decrease in copy number. Three instances of this type of inter-strain variation were found: the *AYT1 *gene (chromosome 12), the *ENB1 *gene (chromosome 15), and the un-characterized ORF *YPL257W *(chromosome 16). The circled regions on the relevant chromosomes in Fig. 3A–D highlight these single-gene strain differences, while Fig. 5A shows the data in more detail for these three genes. *AYT1 *encodes an acetyltransferase that was first characterized in *Fusarium *and subsequently identified in *S. cerevisiae *by homology [75]. It plays a role in de-toxifying endotoxins of the tricothecene family in *Fusarium*, but its function in *S. cerevisiae *is unknown. Although it has been shown that the *S. cerevisiae AYT1 *gene product can acetylate tricothecene *in vivo*, cells that are deficient for this gene show no increased sensitivity to the compound, and in fact show no phenotype at all [75]. *ENB1 *encodes an enterobactin transporter of the major facilitator superfamily involved in iron uptake [81], while *YPL257W *has no known function, although its predicted product has homology to membrane proteins.

### Intra-strain variation

**Figure 4:**
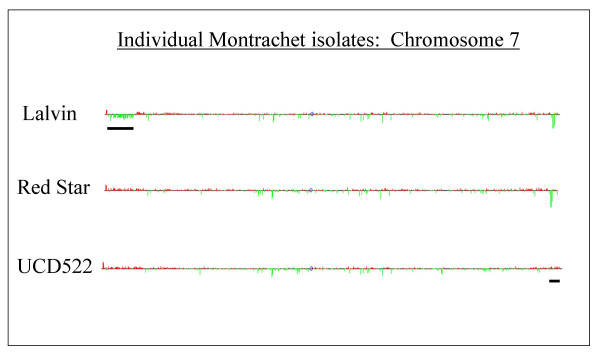
**Karyoscopes of Chromosome 7 from individual Montrachet isolates**. The larger black bar on the left, placed under the left end of chromosome 7 from the Lalvin Montrachet isolate (GSY1), shows a 37-ORF region that has been depleted (or deleted) with respect to the S288C laboratory strain and with respect to the chromosome 7's of each of the other two Montrachet isolates. The smaller black bar on the right, under the UCD522 (GSY3) chromosome, indicates a region of 3 ORFs (including *MAL11 *and *MAL13*) that are present at "wild-type" (S288C) copy number in the UCD522 strain but are depleted or deleted in the other two Montrachet strains. Note that this same *MAL *region is also present at "wild-type" copy number in UCD725, but is depleted or deleted in all remaining wine yeast strains. Again, all karyoscopes are drawn to the same scale.

We observed relatively little genomic variation between independent isolates of each of the different strains. A major exception to this was seen, however, among the three different isolates of Montrachet. As shown in Figure 4, the Montrachet isolate obtained from Lalvin (GSY1) has a fairly extensive region near the left end of chromosome 7 that exhibits copy-number depletion (relative to the S288C reference strain), seen as a distinct cluster of green bars (a thick black line underlines this region). This region spans approximately 37 contiguous genes, all of which show a ratio lower than 1 (shown as green bars). This is in contrast to the other two Montrachet isolates (GSY2 and GSY3 from Red Star and UCD, respectively), which show no copy-number depletion in this region (Fig. 4). Because so many contiguous genes are involved in this block of genes, and because almost all of the genes exhibit a similar direction and magnitude of deviation, we believe that this region has been lost from either one or both copies of chromosome 7 in the (presumably diploid) Lalvin isolate, yielding a net depletion in the copy numbers of these genes relative to the reference strain. It is formally possible that sequence divergence in the region caused the decreased hybridization to the reference strain, but this is unlikely because sequence divergence rarely, if ever, occurs in such a dense and homogeneous pattern, nor is it ever constrained to only one region of the genome. Since there are at least two essential genes in this region (see below), it is most likely that only one of the chromosomes in this presumably diploid strain has lost this region.

It is likely that the region of depletion extends out to the end of the chromosome, despite the fact that the two distal-most genes appear as red (amplified) bars; both genes are sub-telomeric genes that are repeated in the genome and it is thus not possible to determine whether the chromosome 7 copies are specifically present or not. Therefore, the event that caused the depletion of this large region of chromosome 7 in the Lalvin isolate could have been a non-reciprocal translocation resulting in the net loss of the distal end of chromosome 7. This is supported by the fact that the inner (proximal) boundary of the depleted region occurs exactly at a tRNA (tV(AAC)G3). It has been shown that non-reciprocal translocations and other types of gross chromosomal rearrangement events that result in better-adapted genotypes occur frequently during adaptive evolutionary growth, and that these rearrangements are almost always bounded by tRNAs or Ty1 elements [31,56,82-84].

**Figure 5:**
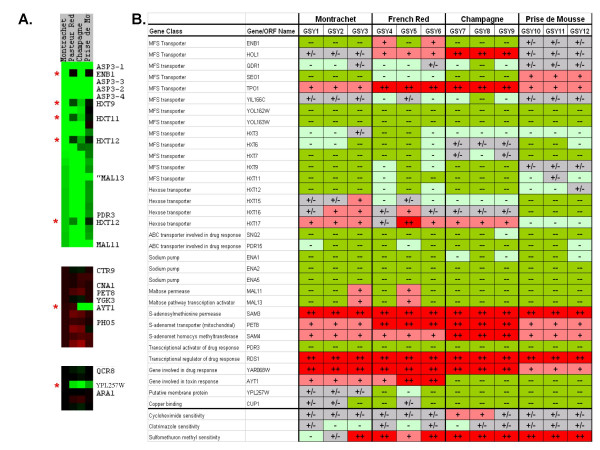
**Unique combinations of genomic differences between different strains. **Panel A: Selected portions of the averaged data shown in Karyoscope form in Figure 3 are shown here in color-block form. To generate this figure the averaged data for all four wine strains were sorted for the Montrachet strain from highest green values (greatest negative number) to highest red values (greatest postitive number) and visualized as a "clustergram" using Java TreeView (note that the data were sorted, but however, were not clustered). The topmost (most green) 25 genes are shown in the top panel; the lower two panels show the sorted data surrounding two of the genes (*AYT1 *in the middle panel, *YPL257W *in the lowest panel) whose copy number varies greatly on a strain-to-strain basis. Red asterisks mark the genes whose copy numbers vary in unique combinations between the different strains. Panel B shows a matrix of gene copy number changes for each of the 12 wine strain isolates, along with the drug response profiles of each strain isolate at the bottom. Within the copy number data portion, a dark red cell with a "++" symbol indicates a red:green log ratio greater than 0.8, light red with "+" indicates a ratio between 0.2 and 0.8, and gray with "+/-" indicates a ratio between 0.2 and -0.2. Likewise dark green with "--" indicates a red:green log ratio less than -0.8, light green with "-" indicates a ratio between -0.2 and -0.8.

The depleted region of Chromosome 7 in the Lalvin Montrachet isolate extends from Open Reading Frame ("ORF") *YGL226W *through *YGL261W*. Included in this region are three genes, two essential and one non-essential, involved in protein-nucleus export (*BRR6*, *CSE1*, *KAP114*), two genes involved in cell cycle control (*SAP4*, *DOC1*), as well as genes involved in secretion (*SEC15*), and respiratory growth (*HAP2*, *MTO1*). Also included in this region is a transporter protein for zinc uptake (*ZRT1*). Of possible significance in terms of winemaking are genes that are involved in glucose/fructose (*HXK2*) [55] and alcohol (*ADH4*) metabolism [85], as well as one gene (*YGL258W*) involved in velum formation in a flor (Sherry) yeast strain. Interestingly, *YGL258W *and *ADH4 *are both induced under zinc-deficient conditions [86,87], which may have significance with respect to the associated depletion of *ZRT1*. If significant fermentative or organoleptic differences exist between the Lalvin Montrachet isolate and the other two Montrachet isolates, it would be interesting to determine whether adding back genes from the depleted region (e.g. on a stable single-copy CEN plasmid) to the Lalvin isolate would cause its fermentative and/or organoleptic characteristics to now mimic those of the other two isolates.

A second intra-strain difference we observed is a depletion of four genes near the right end of chromosome 7 that is seen in both the Lalvin and Red Star Montrachet isolates but not in the UCD522 Montrachet isolate (Fig. 4, shorter black bar underlining the region at the right telomere). This group of four genes includes a maltose permease gene (*MAL11*) and a maltose-activator protein (*MAL13*); loss of these genes usually indicates a defect in maltose fermentation, but they also appear to be involved in drug response pathways since deletions of *MAL11 *lead to nystatin sensitivity [88]. We observed that this same group of *MAL *genes is depleted in all of the other strain isolates except in the French Red isolate UCD725 (GSY5). In other words, of all 12 isolates that we studied, only UCD522 (GSY3) Montrachet and UCD725 (GSY5) French Red do not show a depletion of this *MAL *region relative to the reference S288C strain (Fig. 3A–D, Fig. 5B).

Finally, two genomic differences were seen in the UCD725 French Red isolate (GSY5) when compared to the other two French Red strains (GSY4 and GSY6). First, as mentioned just above, the same group of *MAL *genes that is present at wild-type copy number in the UCD522 Montrachet isolate is also present in this UCD725 isolate, but is depleted in the other two French Red isolates. Secondly, *YPL257W *is depleted in the French Red strains GSY4 and GSY6, but is present at wild-type copy number in GSY5 (data not shown). This gene, encoding a putative membrane protein, is one of the set of genes that exhibit a characteristic copy number genotype between the different wine yeast strains (see below), but it apparently varies in its genotype among the French Red isolates. Overall, however, aside from the fairly small differences among the Montrachet and French Red strains, there is remarkably little intra-strain genomic variation among the strains we studied, indicating that these wine strains are relatively stable genetically, at least as assayed by microarray karyotyping.

### Transporters and permeases are part of the shared group of copy number differences, and also show inter- and intra-strain differences

Figure 5B shows a matrix of relative copy numbers for each of the 12 individual wine strain isolates. The first group of genes shown in the matrix represents all of the Major Facilitator Superfamily (MFS) transporter genes for which a large copy number deviation relative to the reference strain (defined as log_2 _(R/G) value greater than 0.8 or less than -0.8, corresponding to a 1.74 fold change) was seen for at least one isolate. A "-" or "--" sign indicates moderate or large depletion of the gene's copy number, respectively; assuming that these are diploid strains, a "-" would indicate a loss of one of the two copies and a "--" would indicate loss of both copies. Likewise, "+" or "++" indicates moderate or large amplification of the gene, respectively, which would indicate an increase from 2 to 3 copies for "+" and to 4 or more copies for "++". Remarkably, of the total of 27 MFS transporter genes annotated as such in the *Saccharomyces *Genome Database [69,70], almost half (13) show large deviations (as defined above) in copy number among the wine strain isolates; all 13 are shown in Fig. 5B. Most MFS transporters show a depletion in copy number relative to the wild-type strain; *TPO1*, which can transport cycloheximide and other drugs [78], is the major exception, showing amplification in all strains.

Figure 5B also shows copy number data for known permeases and other transporters, as well as any genes annotated as having a role in drug response that show a large deviation in at least one isolate. Finally, genes which do not fall into a transporter/membrane protein class, but which nonetheless stood out in the clustered data as being significantly altered in copy number relative to S288C are included (e.g., the *CUP1 *locus and members of the S-adenosylmethione metabolism pathway). At the bottom of the figure are shown the results of the drug halo assays for each of the isolates (see below).

### Drug resistance/sensitivity phenotypes in wine strains

The prevalence of genes involved in drug response and drug resistance occurring in both the shared group of copy number changes, as well as in inter- and intra-strain differences, directed us to investigate the drug response profiles of the wine yeast strains. We chose three different drugs with which to assay the cells, representing three different general classes of drugs: cycloheximide, "CYH" (a protein synthesis inhibitor); clotrimazole, "CTZ" (member of the azole class of antifungal agents which inhibit ergosterol synthesis); and sulfomethuron methyl, "SMM" (a sulfonylurea herbicide). As described in Methods, halo assays were performed on all 12 wine strains listed in Table 1 as well as on six other control yeast strains mentioned in Methods. These additional six strains represent either control wild-type strains (FY1679, S288C, RW2802) or strains with single-gene deletions of various drug resistance genes whose copy numbers had been observed to be altered in the wine yeast strains: an *AYT1 *deletion strain (ScAYT1Δ; [75]), a *PDR5 *deletion strain (JG436; [75]), and a *YPL257W *deletion strain (GSY28).

**Figure 6:**
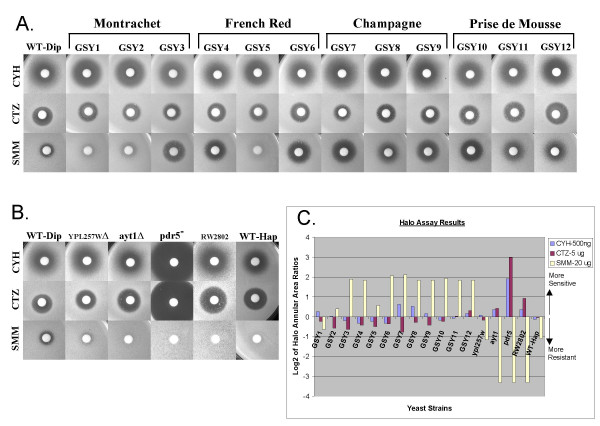
**Drug resistance phenotypes of wine strains**. Halo assays were performed as described in the text to test the response of the wine strains to various drugs. Panels A and B show photographs of the halos; on the far left of each panel are shown the halo assays for the WT-diploid (FY1679, a S288C-based diploid, see Methods), against which all other strains were compared. CYH = 500 ng cycloheximide; CTZ = 5 μg clotrimazole; SMM = 20 μg sulfomethuron methyl. **Panel A: **strains are as described in Methods and Table 1; "WT-Dip" refers to FY1679. **Panel B: **Strains are as described in Methods; "WT-Dip" refers to FY1679; "YPL257 Δ" to GSY28; "ayt1 Δ" to ScAYT1Δ, "pdr5^-^" to JG436; and "WT-Hap" to S288C. **Panel C: **Graphical results of halo assays. Annular area (i.e., area of total halo minus area of disc) was calculated for each halo, then expressed as log_2 _of the ratio of the annular area to that of the WT-Dip (FY1679) annular area.

Figures 6A and 6B show the actual halos on the plates, while Fig. 6C tabulates the halo results in chart form. The most notable feature of the chart is that the majority of the 12 wine strain isolates (Fig. 6A, 6C) are very sensitive to SMM compared to the WT diploid laboratory strain. However, there are three exceptions to the SMM sensitivity, with two of the three Montrachet isolates, Lalvin Montrachet (GSY1) and Red Star Montrachet (GSY2), as well as the UCD725 French Red isolate (GSY5), showing much less sensitivity or even low resistance to SMM.

It can also be seen in Figs. 5B, 6A and 6C that the individual isolates of Champagne (GSY7-9) and Prise de Mousse (GSY10-12) wine strains show more consistency in their drug response profiles than do the individual isolates of Montrachet (GSY1-3) and French Red (GSY4-6). Interestingly, it is the individual isolates of both the Montrachet and the French Red wine strains that also showed the most variability in their genome structures (Fig. 5B).

Halo assays for the control yeast strains are shown in Fig. 6B. As expected, the *pdr5 *strain, known to be hypersensitive to drugs, is extremely sensitive to CYH and to CTZ. It is very resistant to SMM, however, with no halo at all forming around the SMM disc. The *ayt1*Δ strain's response to CTZ or CYH is similar to that of the WT diploid, but like the *pdr5 *strain it is very resistant to SMM. The *YPL257W*Δ strain shows moderate resistance to SMM compared to the WT diploid, with no variant response to CTZ or CYH. Note that both of the haploid wild-type control strains, S288C ("WT-Hap" in figure) and RW2802, show greater resistance to SMM than does the WT diploid strain; S288C is moderately resistant and RW2802 is extremely resistant, with no halo detected in the presence of SMM. This may indicate that haploid strains are for some reason more resistant to SMM than diploid strains. It also appears that strain differences (i.e., compare RW2802 and S288C) may affect the degree of resistance to SMM.

## Conclusion

We have found that each of the three independent isolates of the four commercially-produced wine strains we examined (i.e., 12 strains total) are all *S. cerevisiae *strains, based on their array karyotypes showing that all have genomes highly similar to the standard laboratory *S. cerevisiae *strain (S288C). Two of the wine strains we examined – Prise de Mousse (also known as EC1118 or Premier Cuvée), and Pasteur Champagne – were listed in company catalogs as *S. cerevisiae var. bayanus *or *S. bayanus*, and the other two as *S. cerevisiae var. cerevisiae *or *S. cerevisiae *(e.g., see [89]). Our hybridization data thus show that there is great similarity between *S. cerevisiae var. bayanus *and *S. cerevisiae var. cerevisiae*, supporting the view that variety or race designations within *S. cerevisiae *should be abolished if there is no overwhelming phenotypic or molecular evidence to support their existence; this conclusion was also reached by Fernandez-Espinar et al. in a study comparing different isolates of commercial wine yeasts by restriction fragment polymorphisms, pulsed-field gels, and PCR analysis [90].

Contrary to previous results [23], none of the strains we examined showed any whole chromosome aneuploidies for *S. cerevisiae *chromosomes, although two of the strains we examined (UCD522 and UCD595) were identical to those used in that study. We know from our work with hybrid yeast strains that array karyotyping can easily detect whole chromosome aneuploidies (B. Dunn, unpublished), so we believe that the wine strains we examined in this work indeed do not contain any whole chromosome aneuploidies; we do not know the basis for the discrepancy other than that the previous study used indirect (genetic) means to detect the aneuploidies and it is possible that some of the markers behaved aberrantly.

We also found that the wine strains we examined all shared in common a set of genome copy number changes relative to the standard S288C strain. Many of these changes are either in Ty1 transposons, where copy number changes can be explained by differences in transposition rates, or in tandemly-repeated genes, where gene copy number changes can be explained by unequal cross-overs [35,36]. In fact, copy number depletion relative to the *S. cerevisiae *S288C strain of Ty1 elements and the tandemly-repeated *ASP3 *and *ENA *regions have been noted previously in various non-S288C yeast strains [51,52,57-60], B. Dunn, unpublished); they therefore appear to be changes shared by many, if not all, non-S288C yeast strains; although they are thus not wine strain specific, they are shared by all the wine strains we examined.

We therefore propose a "commercial wine strain signature" that comprises those genes whose copy number is altered in all the wine yeast isolates we examined relative to the sequenced S288C strain, but which excludes any of the genes whose copy numbers are altered in ale or beer strains or in other non-S288C laboratory strains such as SK1 and Y55. The final list of genes in the "commercial wine strain signature" is given in Table 2; it is comprised of a subset of those genes for which a statistically significant change in copy number, either depletion or amplification, occurred in at least 3 of 4 strains, as determined by the CGH-Miner program. All Ty1 genes, the ENA1, 2 and 5 genes, and genes from the ASP3 region on Chromosome XII have been eliminated from this list since they appear to be copy number changes shared by all non-S288C yeast strains and are not wine-specific.

Also of interest are the genes that show inter- or intra-genic variation, shown in Fig. 5B. These genes consist mainly of sets of transporter or sugar utilization genes residing at or near the telomeres. These genes can undergo a type of inter-chromosomal unequal cross-over or gene-conversion event through dispersed sub-telomeric sequences shared among the ends of the chromosomes [91], and thus can undergo changes in copy number relatively facilely. Although many other non-wine, non-S288C strains have been observed to have alterations (predominantly deletions) in sub-telomeric sugar utilization genes [52], none of them show the exact pattern of the "commercial wine strain signature". This is not surprising, since it might be expected that the exact suite of transporter and sugar utilization genes would vary in defined patterns among yeasts, depending on the particular substrate (e.g., grape juice, malted barley, laboratory defined media, or wheat flour) on which they had been selected for good growth performance. In fact, the wine strains are unique in having several sets of sub-telomeric genes, most notably those at the right end of Chromosome I (*YAR064W *– *YAR075W*), amplified relative to the S288C strain. On a blind assessment of a panel of karyoscopes generated from an ongoing study (B. Dunn, unpublished), which included a set of 6 additional commercial wine strains, as well as beer strains (one ale and one lager), laboratory non-S288C strains (Y55 and SK1), and S288C itself, we were easily able to identify the wine strains as a set distinct from the other strains. The assignment as a "wine" strain was based mainly on the presence of the amplified region at the right end of chromosome I, as well as small amplified telomeric regions on both ends of Chromosome III. In addition, a subset of the wine strains exhibited a characteristic depletion of either *AYT1*, *ENB1 *or *YPL257W *as discussed below. We thus believe that the list presented in Table 2 is a *bona fide *"commercial wine strain signature", at least for commercially-produced wine strains; the amplification of the right end of Chromosome I, which has never been reported before, is probably the most defining aspect of the "commercial wine strain signature". These particular genomic changes may be a result of long-term selection for strains that demonstrate good fermentative behavior and good organoleptic characteristics during wine-making. We expect that there may be slight changes made to the list, as more wine yeast array karyotype data are accumulated. We are planning to perform similar studies with "wild" wine yeast strains, as well as with more commercial wine yeasts in order to refine the list, if necessary.

We also observed internally-located single-copy genes (*AYT1*, *ENB1*, *YPL257W*) whose copy numbers vary either as part of the "commercial wine strain signature" or in inter- or intra-strain variation. It is possible that these genes have diverged in their sequences relative to the reference strain to a degree that hybridization no longer occurs, but it is more likely that rather than accumulation of multiple changes localized to just a few isolated genes (which would have to be the result of an extremely strong positive selection) among the whole genome, a more drastic change (either a deletion or amplification) has occurred to the gene. The mechanism by which this might happen is not known but could be explained by the presence of tandem repeats on either side of the gene, thus enabling loop-out or unequal cross-over events.

Many of the changes in genomic copy number in the "commercial wine strain signature", and in inter- and intra-strain differences, involve genes that encode transporter proteins or genes involved in drug resistance phenotypes in laboratory yeasts. Drug sensitivity results for the wine strains show that most are highly sensitive to SMM as compared to the laboratory strain, although it appears that Montrachet strains may not exhibit as high a level of SMM sensitivity as the other strains (Fig. 5B). When the results of the drug sensitivity tests are compared with the copy number genotypes of the transporter, permease, drug response and other variable genes (Fig. 5B), it is apparent that the situation is complex; among all of the genes that vary in copy number in these strains, only one gene's copy number status correlates well with an easily identifiable drug response – *CUP1*, the copper-binding yeast metallothionein gene. It appears that the nine wine strains (i.e., all except GSY1, 2, and 5) which are highly depleted for the *CUP1 *gene (which is tandemly-repeated as 2 copies in the haploid S288C) show hypersensitivity to sulfomethuron methyl (Fig. 5B). Interestingly, the *CUP1 *gene has been implicated as being highly-expressed in an azole-resistant yeast strain [92], indicating that it may play a role in drug response as well as in copper detoxification. Additionally, *pdr13 *deletion mutants are hypersensitive to copper [93]; both studies support our present findings that there is a link between the *CUP1 *locus and drug sensitivity.

There is also a good correlation between the overall observed variability of the copy number genotypes of the wine isolates among each wine strain and the variability (or lack thereof) of the drug responses; the Montrachet and French Red strains showed the most variability in gene copy numbers among their individual isolates, and likewise these two strains show the most variability in the drug response profiles of the isolates (Fig. 5B).

As mentioned above, we observed less inter-strain and intra-strain variation, especially aneuploidies, than would be expected based on previously published studies [23]. However, the strains we studied were all commercial and/or academic strains that are maintained under fairly constant growth conditions, while many of the previous studies showing genome instability and genomic diversity in wine strains were performed with "wild" yeast strains, i.e., those strains that occur spontaneously in uninoculated wine fermentations. It should also be noted that in this study we have analyzed a set of strains that all originated from France. It is possible that as we expand our studies either to wine yeast strains whose origins are outside of this geographic region, or to "wild" yeast strains, there will be greater genomic differences. Nevertheless, specific inter-strain genomic differences among the four wine strains were found that uniquely identify each strain. The three Montrachet isolates are depleted for *ENB1 *and the *HXT9*, *HXT11*, and *HXT12 *genes, but not depleted for *AYT1 *or *YPL257W*. The Prise de Mousse isolates show exactly the opposite characteristics, being depleted for *AYT1 *and *YPL257W*, but not *ENB1 *or *HXT9*, *HXT11*, and *HXT12*. Two of the three Pasteur Red isolates are depleted for *YPL257W*, and all are partially depleted for *HXT9*, *HXT11*, and *HXT12*, but not depleted for *AYT1 *or *ENB1*. Finally, the Champagne isolates are depleted for all of these genes (Fig. 5B). These results suggest the possibility of establishing a database that uniquely identifies every major wine yeast strain based on its array karyotype. This could allow rapid identification of wine strains in, for example, un-inoculated wine fermentations, or in suspected contaminated fermentations, etc., by the comparison of karyotypes from the unknown yeasts to those of the known yeasts.

Some amount of intra-strain variation was found among the French Red isolates and especially in the Montrachet isolates, in which a stretch of 37 genes on the left end of Chromosome VII was depleted in the Lalvin isolate relative to the other two isolates and to the S288C strain; the magnitude of this particular change was somewhat unexpected, and may indicate that wine yeast producers should be more vigilant in periodically checking the genomic integrity of their strains. Microarray karyotyping appears to be a method that can easily be used to check the genomic integrity of such strains.

Overall, however, the amount of both inter- and intra-strain genomic variation was low among the wine yeast strains, indicating firstly that these strains are closely related, and secondly, that they are quite genetically stable. This implies that these strains can generally be propagated under various conditions without fear of large-scale genomic rearrangements occurring. Our characterization of a "commercial wine strain signature", as well as the determination of unique signatures for various of wine yeast strains, may make it possible to use array karyotyping to help winemakers identify unknown yeasts in spontaneous fermentations, to detect contaminating yeasts during fermentation, and to identify the origins of novel yeast strains. Our results also allow the inference that the differences in the fermentation and organoleptic properties ascribed to these different strains may arise from a small number of genetic changes; it must be noted, though, that the microarray karyotyping technique only allows the detection of genetic changes (primarily copy number changes) at the whole-gene level, not single-nucleotide changes, of which there may be many. However, we can easily test, by adding back deleted genes into the genome, e.g., whether the observed copy number differences between the strains do indeed confer the different sensory properties in the finished wine that have been noted by winemakers.

## Methods

### Strains used

Table 1 shows a list of the commercial wine yeast strains used in this study, including the manufacturer ("Source") of each strain. UCD-numbers in parentheses under "Product Name" refer to the Univ. of Calif. Davis Department of Viticulture and Enology strain collection number. All UCD strains were purchased directly from U. C. Davis (Davis, CA, USA), all Lalvin strains were acquired from Vinquiry (Windsor, CA, USA), and all Red Star strains were purchased from "The Wine Lab" (Napa, CA, USA, [94]).

Six additional *S. cerevisiae *strains were used as controls for halo assays:

JG436: *MATa, pdr5::Tn5, leu2, ura3, met5 *(obtained from N. Alexander; [75]);

ScAYT1Δ: *MATa, ayt1*Δ, *ura3, met5 *(obtained from N. Alexander; [75]);

RW2802: *MATa, leu2, ura3, met5 *(obtained from N. Alexander; [75]);

GSY28: *MATa, YPL257W*Δ, *his3*Δ*1, leu2*Δ*0, met15*Δ*0, ura3*Δ*0 *(YeDelStrain #1035 [95]);

FY1679: *MATa/MATα, leu2*Δ*1/+, +/his3*Δ*200, trp1*Δ*63/+, ura3-52/ura3-52 *(S288C-based diploid strain obtained from laboratory of F. Winston; it is the result of a cross between FY23 and FY73; [96]);

S288C: *MATα *(obtained from laboratory of G. Fink).

The S288C strain has been previously described [41] and carries no auxotrophic markers; it represents one of the most widely-used laboratory "wild-type" yeast strains and is also the strain background for the sequenced *S. cerevisiae *strain. The *S. bayanus *strain used in Figure 1 was obtained from Cletus Kurtzman via Tom Pugh; it is the type strain of *S. bayanus *(NRRL Y-12624).

### Microarray karyotyping protocol

In all karyotyping experiments described, we used microarrays onto which had been spotted PCR products corresponding to full-length ORFs from the S288C strain of *S. cerevisiae*, analogous to previously described arrays [97]. The DNA used in the hybridizations was prepared as described [98]. The reference DNA in all experiments was prepared from S288C (the S288C haploid strain; see above). We used S288C as the reference DNA since this is the strain that was sequenced and from which the PCR products used on the microarrays were generated; the Cy3-labeled DNA should thus give a green signal in every channel. After isolation the DNAs were then directly labeled with fluorescently-tagged nucleotides (Amersham, Piscataway, NJ, USA), either Cy3-dUTP for the reference strain or Cy-5 dUTP for the wine strains, using Klenow polymerase (NEB, Beverly, MA, USA) and random hexamers in a random-priming method as described [61]. After labeling, the reactions were heat-inactivated, the experimental (Cy5-labeled) and reference (Cy3-labeled) DNAs were mixed, purified away from unincorporated label using Microcon-30 filters (Millipore, Billerica, MA, USA), and then hybridized to the microarrays at 65°C as previously described [99]. All experiments were performed using duplicate arrays, where we hybridized the same labeling reaction to each of the two arrays. Arrays were scanned with an Axon 4000A scanner and the data were extracted with GenePix (Molecular Devices Corp., Union City, CA, USA) software as described [97]. The data were deposited in the Stanford Microarray Database [62,63] where they can be retrieved for further analysis; the raw data for all microarray hybridizations reported in this paper can also be downloaded from the site: . For all data analysis described, the array data were filtered first to eliminate manually flagged bad spots, then filtered to include spots for which the ratio of green (Cy3) channel mean intensity to Median background intensity was > 1.5 and the ratio of red (Cy5) channel normalized mean intensity to Median background intensity was > 1.0. The filtering was fairly permissive in order to allow truly deleted genes in the wine yeasts (i.e., no red signal at all) to be detected. All data are presented as the average of the values from the duplicate arrays. Karyoscopes were generated by the Java TreeView program [67].

Note that prior to the data filtering described above, all array data were normalized by setting the average log fluorescence hybridization ratio of all array elements to a value of zero; differences in hybridization intensity due to ploidy differences are therefore eliminated. Thus, although most wine strains are of diploid or higher ploidy, the normalization process allows direct comparison to the haploid reference strain so that only changes in gene copy number relative to the haploid state are detected. For example, if a gene has a copy number of two in the WT haploid strain and a copy number of four in the WT diploid strain, the red:green (R/G) hybridization ratio will be 1.0 (and the log ratio will be zero) since the haploid-relative copy number is the same between both strains. If, however, a diploid strain (labeled in red) has experienced a depletion in the copy number so that it now has only two copies of the gene, the R/G hybridization ratio will be 0.5 because the copy number relative to the haploid state has been lowered.

### CGH-Miner

The CGH-Miner program [68] was installed and employed as described in the CGH-Miner User guide and Manual [100]. The karyscope shown in Fig. 2 was generated by the program using the parameters for BAC analysis because it gives the smallest size (three) of the moving-window for data smoothing. Eight separate S288C self-self hybridizations were used as "NN" controls in the CGH-Miner program, giving a robust baseline to determine the shared copy number changes in the wine strains. When the CGH-Miner program [68] generated the consensus plot shown in Fig. 2, it simultaneously created a file showing the genes that are significantly altered in copy number relative to "normal" (self-self) arrays; an abbreviated list of these genes with significantly-altered copy number are given in Table 2.

### Halo assays

For each of the 18 yeast strains described above, 2 ml of liquid YPD medium [98] supplemented with adenine (50 mg/l final) and tryptophan (50 mg/l final) was inoculated with cells from frozen glycerol stocks. The cultures were grown at 25°C overnight until saturated. Cultures were measured for cell density using a Coulter Counter (Beckman-Coulter, Fullerton, CA, USA), then resuspended at 1 × 10^6 ^cells/ml in 4 ml of top agar (= 0.7% agar, 0.82X YPD plus adenine and tryptophan as above) which had been held at 42°C. After quickly vortexing, the mixture was poured smoothly onto YPD+Ade+Trp plates. The top agar was allowed to cool and harden, then 6.5 mm-diameter filter discs pre-loaded with the desired amount of drug were placed onto the surface of the top agar. The amounts of each drug loaded onto the filter discs were: 500 ng cycloheximide (Sigma-Aldrich, St. Louis, MO, USA; 2 μl of 250 μg/ml solution in water), 5 μg clotrimazole (Sigma; 4 μl of 1.25 mg/ml solution in acetone) and 20 μg sulfomethuron-methyl (DuPont, Wilmington, DE, USA; 8 μl of 2.5 mg/ml solution in acetone). Control filter discs containing 8 μl of either water or acetone were also placed on the plates; halos were never observed around these control discs. The plates were then incubated at 25°C for 2 – 3 days after which photographs of the plates were taken. Final halo measurements used for the charts in Fig. 5 were calculated by first measuring the diameter of the total halo (including the central filter disc) and calculating its area, then subtracting the area of the filter disc to give the annular area (i.e., the area of the annulus defined by the outer edge of the disc to the outer boundary of the halo). The annular area for each experimental yeast strain was then divided by that of the strain FY1679 (S288C-based diploid) to yield an annular area ratio for each drug. Finally, the log_2 _of the annular area ratio was calculated and represent the y-axis values shown in Fig. 5B, where positive values indicate greater sensitivity to the drug compared to the WT-diploid control, and negative values indicate greater resistance to the drug compared to WT.

## Abbreviations

CGH: Comparative Genome Hybridization

CTZ: clotrimazole

CYH: cycloheximide

S-AM: S-adenosylmethionine

SGD: Saccharomyces Genome Database

SMM: sulfomethuron methyl

WT: Wild-type

## Authors' contributions

BD planned and designed the experiments, performed the experiments and the data analysis, wrote the main draft of the paper, and generated the figures. RPL aided in the experimental design and in editing the manuscript. GS provided guidance over the entire project, and also aided in figure making, data analysis and editing of the manuscript. All authors read and approved the final manuscript.

## Additional material

The raw data files for all microarray hybridizations presented in this paper may be downloaded at: 
